# Female second-year undergraduate medical students’ attitudes towards research at the College of Medicine, Alfaisal University: a Saudi Arabian perspective

**DOI:** 10.1007/s40037-013-0093-9

**Published:** 2013-11-28

**Authors:** Ahmed Abu-Zaid, Asma Alnajjar

**Affiliations:** College of Medicine, Alfaisal University, PO Box 50927, Riyadh, 11533 Saudi Arabia

**Keywords:** Female, Undergraduate research, Attitudes, Alfaisal University, Saudi Arabia

## Abstract

There is a rapidly increasing movement towards integrating scientific research training into undergraduate medical education. The aim of this study was to explore the perceived attitudes of female second-year undergraduate medical students towards research at the College of Medicine, Alfaisal University, Saudi Arabia, as well as to explore if any differences exist between students with and without previous research experiences. An online, anonymous, cross-sectional, self-rating survey was administered. A two-tailed Mann–Whitney *U* test was used to compare the mean 5-point Likert scale responses between students with and without previous research experiences. Sixty-one students (61/81) participated in the survey with a 75.3 % response rate. Nineteen participants (31.1 %) had previous research experiences. Overall, all students demonstrated positive attitudes towards undergraduate research. There were significant statistical differences in the means of attitudes towards undergraduate research between students with versus without previous research experiences in regards to the following statements: ‘my adequate possession of research knowledge and skills promotes participation in future research activities’ (3.4 vs. 2.9; *p* < 0.02), ‘I will participate in scientific research activities throughout my undergraduate medical education’ (3.7 vs. 3.1; *p* < 0.00), and ‘I have no interest at all in scientific research’ (1.6 vs. 2.4; *p* < 0.01). Previous exposure to scientific research experiences promotes more positive attitudes towards scientific research.

## Introduction

There is a rapidly increasing movement towards integrating scientific research training into undergraduate medical education [1]. Medical schools have different methods of engaging their students in scholarly undergraduate research activities. These methods include research-driven curricula [1], research electives [2], and compulsory research projects for graduation [3]. Students’ development of positive attitudes towards scientific research is a fundamental element of modern undergraduate medical education curricula [4]. However, little is known about how medical students perceive undergraduate research [5].

The aim of this study is to explore the attitudes of female second-year undergraduate medical students towards research at the College of Medicine, Alfaisal University, as well as to explore if any differences exist between students with and without previous research experiences. The rationale for specifically targeting only female students was driven by an observation of two studies that showed female medical students were less interested and engaged in various undergraduate research activities when compared with their male counterparts [6, 7].

## Methods

The study took place at the College of Medicine, Alfaisal University, Riyadh, Saudi Arabia. It is a recently founded (2008), private, non-profit and student-centred College. At the time of the study (academic year 2012–2013), there were only two undergraduate years existing at the College. Only the female second-year undergraduate medical students were eligible to participate in the study. The rationale for excluding the female first-year undergraduate medical students was due to their limited exposure to the basics of scientific research and lack of sufficient understanding and engagement in the College’s various undergraduate research activities.

Participants were requested to voluntarily complete an online anonymous survey using the online instrument SurveyMonkey (SurveyMonkey, Inc., Palo Alto, CA, USA). The survey was administered to assess: (1) participants’ previous research experiences, and (2) their perceived attitudes towards undergraduate research. The survey was peer-reviewed by two senior in-house faculty members and preliminarily pilot tested on a group of students to ensure proper interpretation of responses.

Attitudes towards engagement in scientific research were assessed by the participants’ responses to typical 5-point Likert rating scale statements, as follows: (1-Strongly disagree, 2-Disagree, 3-Neutral, 4-Agree, and 5-Strongly agree). The average 5-point Likert scale responses were presented as means ± standard deviations (SD). A two-tailed Mann–Whitney *U* test was used to compare the average 5-point Likert scale responses between participants with and without previous research experiences. Statistical significance was determined as a *p* value <0.05.

## Results

At the time of the study, there were 81 female second-year undergraduate medical students. Sixty-one students participated in the survey with a response rate of 75.3 %. Only 19 participants (31.1 %) had previous research experiences, all of which (*n* = 19; 100 %) took place during the summer months in the form of summer research mentorship programmes. Thirteen participants (68.4 %) joined local programmes whereas 6 participants (32.6 %) joined international programmes.

Table 1 summarizes the perceived attitudes of the female second-year undergraduate medical students towards research at the College of Medicine, Alfaisal University. Overall, students showed positive attitudes towards undergraduate research and engagement in its activities.

**Table 1:** The perceived attitudes of the female second 0 year undergraduate medical students towards research at the College of Medicine, Alfaisal University

Evaluative statement	Strongly disagree* n* (%)	Disagree* n* (%)	Neutral *n* (%)	Agree *n* (%)	Strongly agree *n* (%)	Mean ± SD (*n* = 61)	Participants with previous research experiences (*n* = 19) Mean ± SD	Participants without previous research experiences (*n* = 42) mean ± SD	*p* value^a^
Training in scientific research is essential to undergraduate medical education	0 (0)	1 (1.6)	16 (26.2)	26 (42.6)	18 (29.5)	4.0 ± 0.8	4.3 ± 0.7	3.9 ± 0.8	0.09
Training in scientific research must be a mandatory component of undergraduate medical education	3 (4.9)	9 (14.8)	22 (36.1)	20 (32.8)	7 (11.5)	3.3 ± 1	3.5 ± 1.0	3.2 ± 1.0	0.28
Scientific research knowledge and skills are relevant in clinical practice regardless of healthcare specialty	0 (0)	2 (3.3)	13 (21.3)	27 (44.3)	19 (31.1)	4.0 ± 0.8	4.2 ± 0.8	4.0 ± 0.8	0.24
I will be involved in scientific research because of an interest in a scientific discipline	0 (0)	6 (9.8)	26 (42.6)	25 (41.0)	4 (6.6)	3.4 ± 0.8	3.5 ± 0.8	3.4 ± 0.8	0.72
I will be involved in scientific research to facilitate entry to residency/fellowship medical programmes	0 (0)	3 (4.9)	13 (21.3)	29 (47.5)	16 (26.2)	4.0 ± 0.8	3.8 ± 1.0	4.0 ± 0.7	0.67
My adequate possession of research knowledge and skills promote participation in future research activities	3 (4.9)	9 (14.8)	32 (52.5)	14 (23.0)	3 (4.9)	3.1 ± 0.8	3.4 ± 1.2	2.9 ± 0.6	0.02^†^
I will participate in scientific research activities throughout my undergraduate medical education	6 (9.8)	3 (4.9)	25 (41.0)	24 (39.3)	3 (4.9)	3.2 ± 0.8	3.7 ± 0.9	3.0 ± 1.0	0.00^†^
I have no interest at all in scientific research	20 (32.8)	20 (32.8)	15 (24.6)	5 (8.2)	1 (1.6)	2.1 ± 1.0	1.6 ± 0.8	2.4 ± 1.1	0.01^†^

^a^A two-tailed Mann–Whitney *U* test was used to compare the mean 5-point Likert scale responses between participants with and without previous research experiences

^†^Statistical significance, *p* value < 0.05

Statistically significant differences of means were identified between participants with and without previous research experiences in regards to the following statements: ‘my adequate possession of research knowledge and skills promotes participation in future research activities’ (mean 5-point Likert scores: 3.4 vs. 2.9; *p* < 0.02), ‘I will participate in scientific research activities throughout my undergraduate medical education’ (mean 5-point Likert scores: 3.7 vs. 3.1; *p* < 0.00), and ‘I have no interest at all in scientific research’ (mean 5-point Likert scores: 1.6 vs. 2.4; *p* < 0.01) (Table 1).

## Discussion

To the best of our knowledge, from a Saudi Arabian perspective, this report serves as the first piece of literature attempting to exclusively inquire into the perceived attitudes of female medical students towards undergraduate research.

At the time of the study, the survey showed that 31.1 % of the female second-year undergraduate medical students at the College of Medicine were involved in some sort of extracurricular undergraduate research initiatives. This percentage (31.1 %) is fairly promising and hopefully expected to increase with the passage of time.

Broadly, female participants showed positive attitudes towards undergraduate research. Almost all participants endorsed the importance (72.1 %) and compulsoriness (44.3 %) of integrating scientific research into undergraduate medical education curricula. These two perceptions were greatly reinforced by the Boyer Commission on Educating Undergraduates in the Research University (1998) [1].

Although it has been demonstrated that participation of students in scholarly research activities can influence the selection of their future medical speciality [8], 77 % of participants affirmed the relevance of scientific research knowledge in clinical practice irrespective of medical speciality. Only a very marginal number of students (9.8 %) expressed no interest at all in participating in undergraduate research experiences—which are considered as extracurricular activities and do not constitute core components of the medical curriculum. Interestingly, the most considerable purpose for partaking in undergraduate research activities was predominately for a ‘strategic’ rationale to facilitate entry to residency/fellowship medical programmes (73.8 %), while less than half of students (47.5 %) expressed engagement in research activities because of an interest in a scientific discipline.

Early participation in undergraduate research activities has been associated with an increased tendency towards further participation in research ventures [9]. Participants with previous research experiences appeared to have more positive attitudes towards further engagement in research endeavours throughout undergraduate medical education than participants without previous research experiences (mean 5-point Likert scores: 3.7 vs. 3; *p* < 0.00). This can be attributed to the fact that in the settings of early introduction to undergraduate research, knowledge of scientific research and familiarity with basic research skills, students will be, to a greater extent, self-assured to carry on further research activities [10]. This was clearly evident in participants with previous research experiences who indicated that acquisition of a good deal of research knowledge and skills would promote participation in future research activities (mean 5-point Likert scores: 3.4 vs. 2.9; *p* < 0.02).

There are several limitations to this study. First, including male second-year undergraduate medical students in the study would have been helpful for valid comparison of research attitudes between females and their male counterparts. Second, this is a self-reporting study, and hence results are liable to recall bias and unverified ratings (overestimation and/or underestimation). Third, the cross-sectional study design necessitates following up participants on the long-term to inquire into changes of their attitudes towards undergraduate research and participation in future research opportunities.

In conclusion, female students with previous research experiences, as opposed to students without previous research experiences, had more positive attitudes towards undergraduate research. Moreover, in our study, perception of ‘female’ students towards undergraduate research is actually difficult to state because of the lack of a comparison with male counterparts or a golden standard. Male students were not included in our study as the focus of study was inappropriately targeted towards female students only. Future research includes investigating the perceptions of both male and female students towards undergraduate research in the various Saudi Arabian local and regional medical colleges. This future research could be an attempt to thoroughly understand the differences across genders, academic levels of education and medical institutions, and accordingly provide a valid generalization status of the perceived attitudes of male and female medical students towards undergraduate research in Saudi Arabia. The findings of this potential study will be compared to the findings of the other regions across the world.
